# Transformation Temperatures and Mechanical Properties in Bending of a Multizone Rhodium-Coated NiTi Archwire: A Retrieval Analysis Study

**DOI:** 10.3390/jfb17030112

**Published:** 2026-02-26

**Authors:** Iosif Sifakakis, Alexandros Banis, Ioulia-Maria Mylonopoulou, Thomai Papadaki, Nikos Boukos, Christoph Bourauel

**Affiliations:** 1 Department of Orthodontics, School of Dentistry, National and Kapodistrian University of Athens, 11527 Athens, Greece; marilimyl@dent.uoa.gr; 2 Electron Microscopy and Nanomaterials Lab, Institute of Nanoscience and Nanotechnology, National Centre for Scientific Research ‘Demokritos’, 15341 Agia Paraskevi, Greece; a.banis@inn.demokritos.gr (A.B.); n.boukos@inn.demokritos.gr (N.B.); 3 School of Dentistry, National and Kapodistrian University of Athens, 11527 Athens, Greece; papthomay@gmail.com; 4 Department of Oral Technology, School of Dentistry, University Hospital Bonn, 53127 Bonn, Germany; bourauel@uni-bonn.de

**Keywords:** multi-zone NiTi archwires, rhodium coating, retrieved, transformation temperature, mechanical behavior, Scanning Electron Microscopy

## Abstract

This study compared the mechanical and thermal properties of new and retrieved multizone rhodium-coated superelastic nickel-titanium (NiTi) archwires across anterior and posterior segments. Using three-point bending tests, Scanning Electron Microscopy with Energy-Dispersive Spectroscopy analysis, and multiple linear regression, it was found that the posterior segments of new wires generated forces 0.50–0.80 N higher than those of anterior or retrieved specimens. While anterior segments exhibited higher austenite start and finish temperatures (by 6.15 °C and 5.21 °C, respectively) compared to posterior segments, these temperatures remained below average intraoral levels, and clinical retrieval did not significantly alter transformation temperatures. However, retrieved wires produced lower overall forces, likely due to surface cracking identified through microscopy. Ultimately, while posterior segments consistently generate higher forces than anterior segments, the observed reduction in force over time and the risk of surface degradation led to the conclusion that these archwires are not recommended for tooth movements exceeding 2 mm.

## 1. Introduction

Nickel–titanium (NiTi) archwires exhibit unique mechanical behavior due to reversible transformations between austenite and martensite phases, with the R-phase sometimes occurring as an intermediate state [1,2,3,4]. This transformation underlies the shape memory effect and occurs within a specific transition temperature range (TTR) [5,6,7]. For orthodontic applications, the TTR must remain below body temperature to ensure reliable superelasticity despite the temperature variations in the oral cavity [8,9,10,11]. Compared with non-heat-activated wires, heat-activated NiTi archwires deliver lighter and more continuous forces [12,13,14,15,16], which is particularly important in the anterior region where excessive forces have been associated with increased susceptibility to root resorption [17].

To address this clinical concern, multizone archwires (NiTi and stainless steel) were introduced in the 1980s [18]. Recent technologies have enabled the development of multizone NiTi archwires capable of exerting lower forces in the midline and higher forces in the molar regions. These wires not only provide region-specific force modulation but also deliver overall lighter forces than conventional superelastic or heat-activated wires with comparable cross-sections [19,20].

Rhodium coating has been applied to NiTi archwires to enhance their esthetic appeal, particularly in visible anterior regions. Recent investigations have shown that while rhodium-coated wires improve visual appearance, the coating may increase surface roughness and reduce nanohardness compared with uncoated NiTi wires, potentially affecting mechanical performance [21,22]. Additionally, rhodium-coated wires demonstrate enhanced corrosion resistance, especially in acidic environments, which may contribute to their longevity and clinical durability [23]. However, some studies have also reported that the color stability of these wires can be compromised by exposure to certain mouthwashes, highlighting potential limitations in their esthetic performance over time [24].

The assessment of thermal properties in heat-activated archwires can be conducted using techniques such as differential scanning calorimetry (DSC), bend-and-recovery (BFR) testing, and magnetization analysis [24,25,26,27]. To ensure consistency across investigations, American Society for Testing and Materials (ASTM) F2082 [25] has been established as the standard for measuring the austenite finish (A_f_) temperature in Nitinol alloys.

This research aimed to assess the mechanical and thermal characteristics of anterior and posterior parts of a rhodium-coated, multizone superelastic NiTi archwire, comparing fresh and intraorally retrieved samples. The null hypothesis proposed that the bending behavior and transformation temperatures would show no differences between the wire segments or between new and used wires.

## 2. Materials and Methods

In the current study, a comparison is made between the following groups of 0.46 × 0.46 mm Rhodium BioActive (GAC, Dentsply Sirona, Long Island City, NY, USA) NiTi archwires: new anterior, new posterior, retrieved anterior, and retrieved posterior samples (LOT E58D, E232).

A total of twenty archwires were evaluated in each group, yielding 40 wires overall. The retrieved specimens were from 20 different patients. Two wire pieces (anterior and posterior) were obtained from each archwire. The specimens were collected after an average of 3.45 (±0.87) months of intraoral use from patients eligible for the study.

The inclusion criteria were (a) ages 11–20, (b) good overall and oral health with a modified Silness and Löe plaque index (PI-M) of 3 or less [28], and (c) non-extraction treatment using fixed appliances in both dental arches for at least 6 months after archwire insertion. Patients with higher PI-M scores or compromised periodontal tissue were excluded. An equal number of male and female patients were included in the sample.

The study was approved by the Ethics Committee of the School of Dentistry, National and Kapodistrian University of Athens, Greece (approval code: 696/06.03.2025, approval date: 6 March 2025). Informed consent for participation was obtained from all subjects involved in the study.

### 2.1. 3-Point Bending Test

The Orthodontic Measurement and Simulation System (OMSS) was employed to conduct the 3-point bending tests. This setup includes two motor-driven positioning stages and two force/moment sensors capable of detecting forces and moments in three dimensions. These sensors connect to a personal computer with a custom control program that records movements while measuring multidimensional force–deflection (N/mm) or moment–degree curves (Nmm/degree). The force measurement range is ±15.00 N, and the moment range is ±450 Nmm. During cycling testing, the OMSS is placed inside a temperature-controlled chamber maintained at 37 ± 1 °C (VEM 03/400 Vötsch Heraeus, Weiss Technik GmbH, Reiskirchen, Germany) [29].

The wire sections from the anterior (midline) and posterior (molar region) areas were cut into 20 mm pieces and prepared for the three-point bending test. The support point distance was 10 mm, as specified by DIN EN ISO 15841 [30]. The wire pieces reached a maximum deflection of 3.3 mm when pushed by the centered thrust die between the support points. Measures were implemented to prevent slipping or flipping. Loading and unloading were performed at a rate of 0.1 mm/s, with no dwell time between phases. Testing was conducted with the archwires in the edgewise orientation. Each cycle was executed twice on each of the 20 wire samples, and the mean value was utilized for statistical analysis. The force/deflection curve was recorded using the OMSS. Forces (in N) during the unloading phase were measured and analyzed at deflections of 3.1 mm, 3.0 mm, 2.0 mm, and 1.0 mm.

The bending moduli (*Eb*) were calculated from the linear part of the 3-point bending force–deflection curves using the following formula:

(1) $$Eb=\;\frac{L^{3}\Delta F}{4wh^{3}\Delta d}$$

where Δ*F* and Δ*d* represent the differences in force and deflection, respectively, between the unloading points. *L* refers to the support span (10 mm), *w* indicates the width (measured in millimeters), and *h* signifies the archwire height (in millimeters). The deflection loads were obtained from the data within the linear segment of the unloading curve, between 0.1 and 0.5 mm. This range was selected as it corresponds to the bending moment of the austenite region.

### 2.2. Bend-and-Free Recovery Test (BFR)

Bend-and-free recovery tests following ASTM F 2082/F 2082M–2023 [25] were performed to identify the critical transformation temperatures of austenite start (A_s_) and finish (A_f_). The specimens were cooled in a custom-made, double-walled glass container below the A_f_ temperature, where a fully martensitic microstructure is anticipated. They were then deformed, supported on two pins, and reheated using an F12-ED (Julabo, Seelbach, Germany) to restore the fully austenitic phase [31]. The wire was cooled in a cold mix bath (Centro Kühlerschutz, Karlsruhe, Germany, >50% 1,2-ethanediol) to at least −20 °C, and submerged for a minimum of 3 min prior to testing. The measurement began as the thermostat was turned on and heating was gradually increased. A laser triangulation sensor (RF603, Riftek Sensors & Instruments, Minsk, Belarus) tracked the specimen’s displacement versus temperature, allowing determination of A_s_ and A_f_ from the displacement–temperature graph. Data on displacement and temperature were recorded throughout heating and recovery, then saved as text files and imported into Microsoft Excel to generate graphs. For each wire, a temperature–displacement graph was created and a tangent line was drawn on the linear sections of the curve. Only the data points on the last section (linear) of the graph were used to draw the line with the trendline function of the software.

### 2.3. SEM/EDS Analysis

The surface morphology and elemental composition of new specimens were studied with a Quanta Inspect Scanning Electron Microscope (FEI, Hillsboro, OR, USA) and by energy-dispersive X-ray microanalysis (EDS) employing a PV7760/68 ME detector (EDAX AMETEK, Warrendale, PA, USA) under high vacuum, 25 kV accelerating voltage, and 98 μA beam current, operating in Secondary Electron (SE) imaging mode. For the elemental analysis, a 200 s acquisition time and 25–35% dead time were selected. Quantitative analysis of collected spectra was carried out by the dedicated software (EDAX Genesis, V4.5, AMETEK) employing ZAF (atomic number, absorbance, fluorescence) correction factors.

### 2.4. Sample Size Calculation

Sample size calculation was based on a recent in vitro experiment that measured force magnitude and differences between the anterior and posterior segments of multizone NiTi archwires [19]. The smallest relevant effect was observed in the unloading forces at 2 mm. At this point, which marked the onset of a horizontal unloading plateau, the mean posterior–anterior difference was 0.20 N. This value was therefore used as the minimal detectable difference for the sample size calculation and was assumed to have a conservative standard deviation of paired differences (±0.05 N), yielding a standardized effect of dz ≈ 0.75. The required numbers of pairs under the above-mentioned assumptions are 14 pairs for 80% power and ≈19 for 90% power. To ensure adequate precision across endpoints and allow for potential data loss, a conservative target of 20 samples was adopted, which exceeds the calculated requirements and allows model diagnostics. This sample size was further confirmed using G*Power 3.1 (Windows), yielding a statistical power exceeding 95% (https://www.psychologie.hhu.de/arbeitsgruppen/allgemeine-psychologie-und-arbeitspsychologie/gpower, accessed on 25 November 2025). A recent study comparing new and retrieved multizone rectangular superelastic NiTi archwires found no statistically significant difference at 2 mm [−0.03 N (−0.10, 0.05)] [31].

### 2.5. Statistical Analysis

Statistical analysis and data management were carried out using IBM SPSS Statistics 29, while visualization was done in Python with libraries such as pandas, statsmodels, matplotlib, and seaborn. To explore potential differences between measurements based on wire type (new or retrieved) and segment (posterior or anterior), a multiple linear regression model with a random intercept at the wire level was employed, using wire type and measurement location as covariates. The dependent variables included (1) unloading force (F, in N) at each displacement point; (2) bending modulus (Eb, in GPa); and (3) transformation temperatures (A_s_ and A_f_, in °C). Interactions between wire type and segment were also examined; the model incorporating interactions was chosen if the interaction term was statistically significant. All analyses were two-tailed, with a significance level of α = 0.05.

## 3. Results

### 3.1. 3-Point Bending Test

Posterior segments delivered higher unloading forces than anterior segments across all activation points (Table 1 and Table 2). On new wires, the posterior–anterior mean differences reached 0.77 N (95% CI 0.70–0.83) at 1 mm, 0.71 N (95% CI 0.65–0.78) at 2 mm, 0.50 N (95% CI 0.43–0.57) at 3 mm, and 0.45 N (95% CI 0.37–0.53) at 3.1 mm. Retrieved wires maintained the same hierarchy, with posterior forces surpassing anterior forces by 0.60 N (95% CI 0.54–0.66), 0.64 N (95% CI 0.59–0.69), 0.53 N (95% CI 0.48–0.59), and 0.55 N (95% CI 0.49–0.61) at 1.0, 2.0, 3.0, and 3.1 mm, respectively. Retrieved wires attenuated unloading forces regardless of segment: retrieved wires delivered 0.17 N (95% CI 0.10–0.23) less force than new wires on the anterior segment at 1 mm and 0.34 N (95% CI 0.28–0.40) less on the posterior segment; the reductions remained between 0.20 N and 0.28 N at 2 mm, and 0.22–0.25 N at 3 mm for both segments, before converging to 0.27 N (anterior) and 0.17 N (posterior) at 3.1 mm (Table 3).

**Figure 1:**
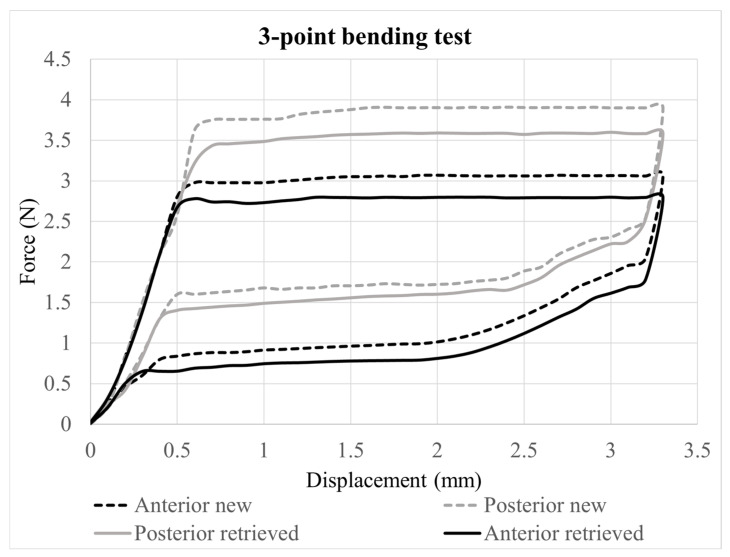
The 3-point bending curves of new and retrieved Bioactive archwires; linear unloading regions were used to calculate Eb.

Representative curves of anterior and posterior wire sections from the 3-point bending tests are depicted in Figure 1.

The Eb of new wires on the posterior segments of the archwire were higher than the anterior segment by 1.4 GPa (95% CI 0.3–2.5) and by 1.1 GPa (95% CI −0.3–2.5) in retrieved wires. The Eb of retrieved wires were lower by 0.8 GPa (95% CI −1.9 to 0.4) on the anterior segment and by 1.1 GPa (95% CI −2.4 to 0.2) on the posterior segment when compared to new wires. Only the difference between anterior and posterior segments in new specimens was statistically significant (*p* = 0.012) (Table 1 and Table 4).

### 3.2. Bend-and-Free Recovery Test (BFR)

The transformation temperature A_s_ obtained from the anterior segment was on average higher by 6.15 °C (95% CI: −6.85 to −5.44, *p* < 0.001) on new wires and by 5.72 °C (95% CI: −6.52 to −4.92, *p* < 0.001) on retrieved wires, compared to the posterior segments. Similarly, A_f_ obtained from the anterior segment was higher by 5.21 °C (95% CI: −6.09 to −4.32, *p* < 0.001) and by 4.67 °C (95% CI: −5.48 to −3.86, *p* < 0.001) on new and retrieved wires, respectively (Table 5 and Table 6).

**Figure 2:**
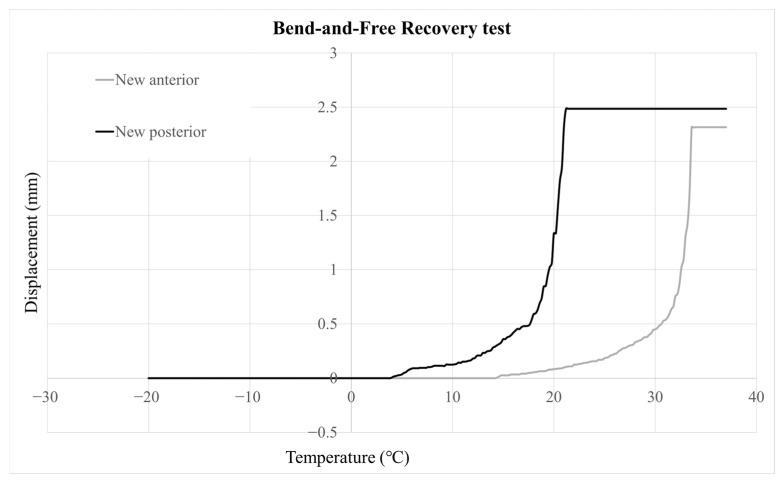
Representative temperature−displacement graphs of Bioactive archwires (posterior and anterior sections) from the BFR test.

The differences in A_s_ and A_f_ temperatures between new and retrieved wires were not statistically significant after adjusting for measurement location. Representative displacement–temperature graphs of anterior and posterior wire sections are depicted in Figure 2.

### 3.3. SEM/EDS Analysis

**Figure 3:**
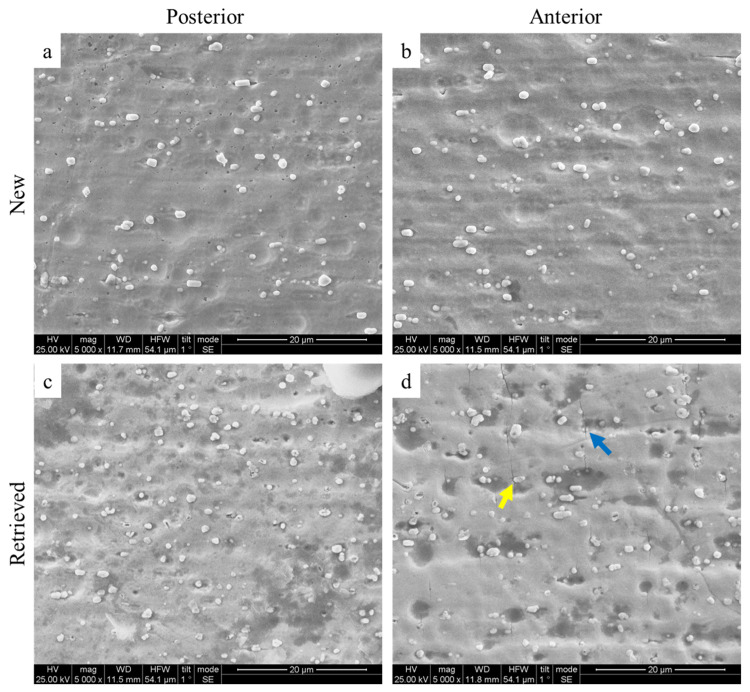
Representative Secondary Electron (SE) images from the surface of the new (**a**,**b**) and the retrieved (**c**,**d**) archwires. Both posterior and anterior segments are provided. The image is oriented with the wire’s long axis parallel to the horizontal axis (nominal magnification 5000×). The yellow arrow indicates a crack that propagates around the rhodium particles, and the blue arrow shows a crack that shears through a rhodium particle.

Figure 3 illustrates representative SEM images from the anterior section of both the new and retrieved specimens. In both cases, the archwire surface appears similar, characterized by globular particles uniformly distributed throughout its thickness.

**Figure 4:**
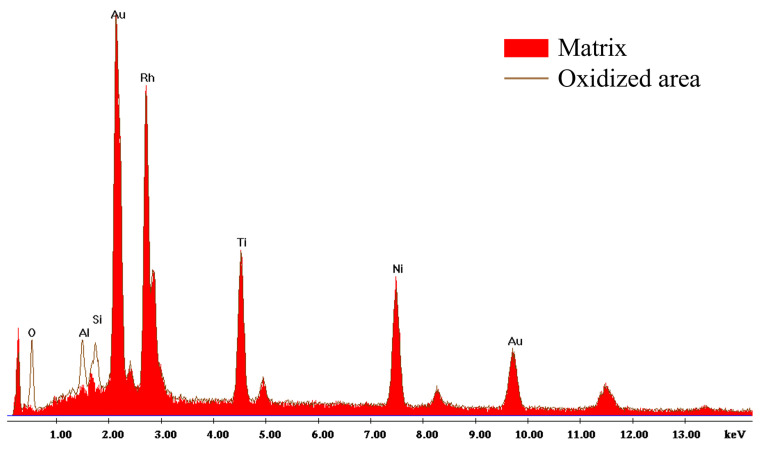
Representative EDS spectrum from the retrieved archwire, taken from the matrix area and from the oxidized area that appears darker.

EDS analysis (Figure 4, Table 7) indicates that these particles contain a higher proportion of rhodium compared to the surrounding matrix. Darker regions on the surface of the retrieved wires suggest oxidation, as indicated by increased oxygen content detected by EDS. Furthermore, the anterior portion of the retrieved archwires exhibits a significant number of cracks, predominantly oriented perpendicular to the wire’s length. These cracks appear to either shear the rhodium particles (blue arrow) or propagate around them (yellow arrow). The cracks are not observed in the posterior segment of the retrieved archwire.

## 4. Discussion

The null hypothesis was rejected: as-received and retrieved specimens of Rhodium BioActive NiTi orthodontic archwires showed differences in specific thermal and mechanical properties between their posterior and anterior sections.

### 4.1. Mechanical Properties in Bending

The three-point bending test is a simple, standardized method (ISO 15841 [30]) used to determine a material’s flexural properties, and the present configuration allows comparison with previous investigations [11,20,31]. In the present investigation, the posterior segments consistently generated forces 0.50–0.80 N higher than the anterior segments at all deactivation points, possibly due to differences in cold-working during manufacturing or wire composition. The multiple force zones observed in the tested NiTi wires align with the recent literature [11,20,31].

The 0.46 × 0.46 mm archwires used in this study generated lower forces than the 0.41 × 0.56 mm NiTi Bioforce wires previously examined [31]. In rectangular wires, width directly affects stiffness and strength, while stiffness depends on the third power of thickness and strength on the square of thickness, so the smaller 0.41 mm thickness reduces mechanical resistance [32]. Mechanical properties may also vary with alloy composition [33]. Limited evidence exists on 0.46 × 0.46 mm NiTi archwires; one study using a different test setup found that 0.46 × 0.64 mm CuNiTi wires were only 15% stiffer than 0.46 × 0.46- mm wires of the same alloy at 1 mm deflection [34].

In the present configuration, the forces in the initial two deactivation stages (3 and 3.1 mm) and from posterior segments at 2 mm exceeded 1 N. A recent systematic review identified 0.1–1 N as optimal for bodily tooth movement [35], with variation depending on the type of movement [36]. Therefore, the tested 0.46 × 0.46 mm rhodium-coated NiTi multizone wires may be unsuitable for tooth displacements greater than 2 mm in clinical situations resembling the present configuration.

Earlier studies suggested that heat treatment before rhodium coating could change mechanical properties [37]. More recent research shows that rhodium-coated 0.46 mm heat-activated NiTi wires exert higher forces than epoxy-coated or uncoated wires [38]. In this study, retrieved specimens exhibited lower forces; although the reason is unknown, prior research reports irreversible austenite-to-martensite transformation, reduced hysteresis energy [39], lower fatigue cycles [40], and possible hydrogen absorption related to fretting-corrosion [41], although another study disputes hydrogen as the main cause of failure [42].

### 4.2. Bend-and-Free Recovery Test

This study offers new insights into the mechanical and thermal behavior of rhodium-coated NiTi archwires. Using the bend-and-free-recovery (BFR) method [43], consistent regional differences in transformation temperatures were observed: anterior segments exhibited higher A_s_ values by 6.15 °C in new wires and 5.72 °C in retrieved wires, and higher A_f_ values by 5.21 °C and 4.67 °C, respectively. After accounting for measurement location, clinical aging did not lead to significant thermal changes. Similar BFR-based regional variation among different wire sizes has been reported [43].

DSC studies of multiforce NiTi, including rhodium-coated wires, show even broader A_s_ and A_f_ ranges, with rhodium-coated types exhibiting the highest A_s_ values [44]. Lower temperatures reported in DSC work may stem from methodological differences, cross-sectional variation, or strain effects. Temperature discrepancies of 4–6 °C between BFR and DSC have been documented [43], and transformation temperatures increase with increasing strain [44].

For optimal superelasticity, the transformation range should remain below the average intraoral temperature [45]. Superelasticity begins once A_s_ is exceeded and is complete after A_f_ [8], but current results suggest that anterior segments may not be fully austenitic intraorally, especially when activated, which could contribute to temperature-dependent force changes. Steeper BFR slopes in posterior segments indicate a faster thermally driven transformation there.

The transformation pathway is further influenced by the R-phase, which may serve as an intermediate structure [46]. In superelastic alloys, R-phase formation begins below 0 °C, whereas non-superelastic wires remain mostly martensitic at room temperature [46]. DSC studies often find it hard to identify A_s_ because R-phase completion overlaps with the start of austenitic transformation [43]. Consistent with these findings, previous work reported minimal changes in transformation behavior after intraoral use. This DSC study on CuNiTi wires found no significant differences between new and retrieved specimens, except for a reduced heating enthalpy at 27 °C in a few samples [47].

Intraoral temperatures typically range from 33 to 37 °C. Because NiTi fully exhibits superelasticity only after A_f_ is exceeded, force levels may fluctuate when hot or cold beverages are consumed if A_f_ exceeds mouth temperature [11]. In this study, the A_f_ of rhodium-coated wires remained below the average intraoral temperature.

### 4.3. SEM/EDS Analysis

SEM images revealed surface cracks on the anterior part of the retrieved archwires. These cracks may result from cyclic loading caused by continuous, repetitive forces during mastication or orthodontic treatment [40,48,49]. Furthermore, surface degradation, indicated by dark-oxidized areas, could be a primary factor in crack initiation and propagation [50,51].

Surface cracks, like those seen in the anterior segments of the retrieved archwires, act as stress concentrators. When a load is applied, this can lead to early failure. As a result, these cracks impair the archwire’s performance during bending tests. This also explains why the posterior segments exhibit higher magnitude forces than the anterior segments, which lack surface cracks. No cracks were observed in the new archwires; hence, they exert higher forces.

These findings align with recent research on the fracture resistance of both as-received and retrieved NiTi archwires. All retrieved wires showed a decrease in the number of cycles to failure. SEM analysis indicated that fractured surfaces were smoother in round cross-section retrieved wires [40].

### 4.4. Limitations

Despite the valuable insights obtained, this study has certain limitations that should be considered when interpreting its findings. The investigation relied on standardized in vitro mechanical and surface assessments, which cannot fully replicate the complex intraoral environment (saliva, plaque, food debris, corrosion, temperature, and mastication) [51]. Factors such as fluctuating temperature, cyclic loading, chemical exposure, and biological interactions may influence the behavior and long-term performance of NiTi archwires differently in vivo. In addition, only specific wire dimensions and brands were evaluated, which may limit the generalizability of the results to other commercial products [31]. Future studies incorporating a wider variety of archwire types and employing in vivo or clinically simulated models would provide a more comprehensive understanding of NiTi wire behavior under real orthodontic conditions.

## 5. Conclusions

- In 0.46 × 0.46 mm rhodium-coated BioActive archwires, 3-point bending tests showed that posterior segments exerted higher forces than anterior segments in both new and retrieved specimens (used intraorally for 3.5 months).
- A decrease in force magnitude was observed in retrieved (of both sexes in equal proportion) specimens relative to new ones. Qualitatively, this decline coincided with visible surface cracking, suggesting that intraoral wear may influence the material’s mechanical integrity.
- The austenite finish (A_f_) temperature of these archwires lies below the average intraoral temperature, indicating that they fully exhibit superelasticity in the mouth.
- Based on these laboratory-based findings, these archwires may not be suitable for tooth displacements of ≥2 mm.

## Figures and Tables

**Table 1:** Mean and standard deviation of force (N) in anterior and posterior segments of new and retrieved multizone NiTi archwires.

Measurement Variables	New Wires Anterior	New Wires Posterior	Retrieved Wires Anterior	Retrieved Wires Posterior
3-point bending: 3.1 mm (N)	1.96 ± 0.08	2.40 ± 0.14	1.68 ± 0.06	2.24 ± 0.11
3-point bending: 3 mm (N)	1.86 ± 0.08	2.36 ± 0.13	1.61 ± 0.05	2.14 ± 0.11
3-point bending: 2 mm (N)	1.01 ± 0.10	1.73 ± 0.09	0.81 ± 0.06	1.45 ± 0.08
3-point bending: 1 mm (N)	0.91 ± 0.13	1.68 ± 0.06	0.75 ± 0.07	1.34 ± 0.11
Eb (GPa)	34.7 ± 1.9	36.1 ± 1.4	33.9 ± 1.7	35.0 ± 2.5
A_s_ (°C)	32.50 ± 0.55	26.35 ± 1.19	32.10 ± 1.05	26.38 ± 1.09
A_f_ (°C)	34.61 ± 0.64	29.41 ± 1.50	34.15 ± 0.92	29.47 ± 1.21

**Table 2:** Results of 3-point bending tests in the posterior vs. the anterior segments: mean difference in N (MD) of measurements and 95% CI of the difference (posterior minus anterior measurements).

Measured Variables	MD Between Posterior–Anterior (N)	95% CI	*p*-Value
3.1 mm activation (new)	0.45	(0.37, 0.53)	<0.001
3 mm activation (new)	0.50	(0.43, 0.57)	<0.001
2 mm activation (new)	0.71	(0.65, 0.78)	<0.001
1 mm activation (new)	0.77	(0.70, 0.83)	<0.001
3.1 mm activation (retrieved)	0.55	(0.49, 0.61)	<0.001
3 mm activation (retrieved)	0.53	(0.48, 0.59)	<0.001
2 mm activation (retrieved)	0.64	(0.59, 0.69)	<0.001
1 mm activation (retrieved)	0.60	(0.54, 0.69)	<0.001

**Table 3:** Results of 3-point bending tests comparing retrieved and new specimens: mean difference in N (MD) and the 95% CI of the difference (retrieved minus new).

Measured Variables	MD Between Retrieved-New (N)	95% CI	*p*-Value
3.1 mm activation (anterior)	−0.27	(−0.32, −0.22)	<0.001
3.1 mm activation (posterior)	−0.17	(−0.25, −0.09)	<0.001
3 mm activation (anterior)	−0.25	(−0.29, −0.20)	<0.001
3 mm activation (posterior)	−0.22	(−0.29, −0.14)	<0.001
2 mm activation (anterior)	−0.20	(−0.25, −0.15)	<0.001
2 mm activation (posterior)	−0.28	(−0.34, −0.22)	<0.001
1 mm activation (anterior)	−0.17	(−0.23, −0.10)	<0.001
1 mm activation (posterior)	−0.34	(−0.40, −0.28)	<0.001

**Table 4:** Mean ± 95% CI of elastic modulus (Eb) in anterior and posterior segments of new and retrieved wires.

Wire Status	Segment	Mean Eb (GPa)	95% CI
New	Anterior	34.7	(33.8, 35.6)
New	Posterior	36.1	(35.4, 36.8)
Retrieved	Anterior	33.9	(33.1, 34.7)
Retrieved	Posterior	35.0	(34.0, 36.0)

**Table 5:** Results of bend-and-free recovery tests: mean difference (MD) in degrees Celsius and 95% Confidence Interval (CI) of measurements.

Transformation Temperature Type	Segment	Mean (°C)	Difference Posterior–Anterior (°C)
A_s_	Anterior	32.50	−6.15
A_s_	Posterior	26.35	
A_f_	Anterior	34.61	−5.21
A_f_	Posterior	29.41	

**Table 6:** Results for the transformation temperatures (in degrees Celsius, °C) in posterior vs. anterior and new vs. retrieved specimens: mean difference (MD) of measurements and 95% Confidence Interval (CI) of the difference.

	MD Between Posterior–Anterior	95% CI	*p*-Value	MD Between New–Retrieved	95% CI	*p*-Value
A_s_ (°C)	−5.72	(−6.52, −4.92)	<0.001	0.40	(−0.23, 1.03)	0.202
A_f_ (°C)	−4.67	(−5.48, −3.87)	<0.001	0.47	(−0.13, 1.06)	0.119

**Table 7:** EDS quantification showing atomic percent (at. %) of elements on the archwire surface.

Element	Matrix	Oxidized Areas	Rhodium Particles
Rh L	31.6	17.5	43.5
Ti K	20.6	11.2	16.2
Ni K	24.9	13.5	17.2
Au L	18.6	10.7	17.9
O K	1.1	37.5	2.4
Al K	1.9	5.3	1.5
Si K	1.1	4.2	1.3

## Data Availability

The original contributions presented in the study are included in the article, further inquiries can be directed to the corresponding author.
